# Advances in the Diagnosis and Treatment of Enthesitis-Related Arthritis

**DOI:** 10.3390/children10101647

**Published:** 2023-10-02

**Authors:** Simona Di Gennaro, Gennaro Di Matteo, Gianmarco Stornaiuolo, Federica Anselmi, Teresa Lastella, Francesca Orlando, Maria Alessio, Roberta Naddei

**Affiliations:** 1 Section of Pediatrics, Department of Translational Medical Sciences, University of Naples Federico II, 80131 Naples, Italy; simona.digennaro95@gmail.com (S.D.G.); gennarodimatteo1988@libero.it (G.D.M.); g.stornaiuolo26@gmail.com (G.S.); teresa.lastella87@gmail.com (T.L.); alessio@unina.it (M.A.); 2 Unit of Pediatric Infectious Diseases, Mother and Child Department, University Hospital Federico II, 80131 Naples, Italy; federica.anselmi@unina.it; 3 General Pediatrics and Immuno-Rheumatology Unit, Santobono-Pausilipon Hospital, 80129 Naples, Italy; f.orlando@santobonopausilipon.it

**Keywords:** enthesitis-related arthritis, juvenile idiopathic arthritis, childhood arthritis, pediatric rheumatology, sacroiliitis, juvenile spondyloarthritis

## Abstract

Enthesitis-related arthritis (ERA) represents 5–30% of all cases of juvenile idiopathic arthritis (JIA) and belongs to the spectrum of the disorders included in the group of juvenile spondyloarthritis. In the last decade, there have been considerable advances in the classification, diagnosis, monitoring, and treatment of ERA. New provisional criteria for ERA have been recently proposed by the Paediatric Rheumatology INternational Trials Organisation, as part of a wider revision of the International League of Associations for Rheumatology criteria for JIA. The increased use of magnetic resonance imaging has shown that a high proportion of patients with ERA present a subclinical axial disease. Diverse instruments can be used to assess the disease activity of ERA. The therapeutic recommendations for ERA are comparable to those applied to other non-systemic JIA categories, unless axial disease and/or enthesitis are present. In such cases, the early use of a TNF-alpha inhibitor is recommended. Novel treatment agents are promising, including IL-17/IL-23 or JAK/STAT pathways blockers.

## 1. Introduction

Juvenile idiopathic arthritis (JIA) is an umbrella term encompassing arthritis of unknown etiology, lasting for >6 weeks and with onset at <16 years of age [1,2]. JIA is the most common chronic rheumatic disorder of childhood, with an incidence rate ranging from 1.6 to 23/100,000 [3]. Enthesitis-related arthritis (ERA) is one of the seven JIA subtypes outlined by the International League of Associations for Rheumatology (ILAR) classification for JIA [4] (Table 1).

Together with the ILAR categories of juvenile psoriatic arthritis (JPsA) and some forms of undifferentiated arthritis, ERA also belongs to the broader group of childhood spondyloarthritis (SpA), a family of inflammatory rheumatic disorders that have some key features in common, including peripheral arthritis, axial involvement, and enthesitis.

In recent years, there have been considerable advances in terms of proposed ERA classification, early recognition of axial involvement, disease activity assessment, and treatment options for ERA.

Therefore, in this review, we outline the historic evolution of ERA definition and classification, describe its clinical spectrum and the disease activity monitoring tools, and summarize the current treatment recommendations and the emerging therapeutic options.

## 2. Methods

An extensive PubMed search of full text English articles was performed to carry out this narrative review. The terms ‘enthesitis-related arthritis’ or ‘juvenile spondyloarthritis’ were used as keywords. We excluded articles published in non-peer reviewed journals. No date filters were adopted, although the authors’ attention was mainly centered on the literature of the last five years (2017 to date). At least two distinct authors reviewed separately the selected articles. Papers considered more elucidative were withheld. The references of relevant articles were also examined to find other possible appropriate sources. Reviews and book chapters have been cited to give readers additional information.

## 3. Historic Evolution of ERA Definition and Classification

To delve into the definition of ERA, it is important to be familiar with the notion of SpA. In 1974, Moll and coworkers introduced the expression “seronegative spondyloarthritis”, to encompass a group of inflammatory arthritis with overlying clinical features and shared genetic predisposition [6]. Common clinical characteristics of SpA include axial involvement (sacroiliitis and/or spondylitis), peripheral arthritis, and enthesitis. SpAs are distinguished by the absence of rheumatoid factor (RF) and by a strong association with the human leukocyte antigen–B27 (HLA-B27) [7]. Some extra-articular features may be present, such as acute anterior uveitis, psoriasis, and inflammatory bowel disease (IBD). Certain clinical characteristics of SpA tend to cluster in some disease subgroups, including ankylosing spondylitis (AS) [8], psoriatic arthritis, enteropathic arthritis, and reactive arthritis. The term “undifferentiated SpA” has been used to describe patients with SpA who do not fit criteria for one of these subgroups [9,10]. To date, adult SpAs are classified according to the two sets of the Assessment of SpA International Society (ASAS) criteria, one for patients with axial involvement and one for those with peripheral symptoms [11,12,13]. Axial SpA (axSpA) can be distinguished in radiographic axSpA, which requires the presence of definite radiographic axial disease and corresponds to AS, and non-radiographic axSpA [12]. ASAS criteria also include reactive arthritis, arthritis related to IBD, and psoriatic arthritis within SpA, encompassing the broad spectrum of disease, although the ClASsification Criteria for Psoriatic ARthritis (CASPAR) are the most used for the classification of psoriatic arthritis in adulthood [11,12,13,14].

Historically, pediatric patients with clinical features resembling adult SpA have been described using different terms: juvenile SpA (JSpA), juvenile ankylosing spondylitis (JAS), seronegative enthesopathy and arthropathy syndrome (SEA), and ERA [15].

The term JSpA refers to patients who develop arthritis in late childhood and adolescence having a robust association with HLA-B27 and potential axial disease [16], whereas JAS includes children and adolescents having peripheral and axial arthritis that fit the criteria for classification of adult AS [17]. Rosenberg and Petty introduced the term “SEA” in 1982 to describe a peculiar form of juvenile SpA in 39 children with arthritis and enthesitis who were RF- and antinuclear antibody (ANA)-negative [18]. According to the authors, SEA was distinguishable from both juvenile rheumatoid arthritis as defined at the time by the American College of Rheumatology (ACR) [19], and from JAS. Although patients with SEA syndrome had enthesitis together with peripheral arthritis, they did not meet the criteria for AS due to the lack of axial involvement, at least at the onset [8,18].

As an evolution of the concept of SEA, the term ERA was introduced in the mid 1990s, when the ILAR proposed a set of criteria for the classification of idiopathic arthritis of pediatric age [20], revised in 1997 [21] and then in 2001 [4], using the umbrella term of JIA and outlining seven disease subtypes (Table 1). According to the ILAR criteria, ERA is defined as arthritis and enthesitis lasting for at least 6 weeks in a child younger than 16 years, or arthritis or enthesitis plus two of the following: sacroiliac tenderness or inflammatory lumbosacral pain; HLA-B27 presence; onset of arthritis in a male older than 6 years; acute (symptomatic) anterior uveitis; and family history of a HLA-B27-related disorder (Table 2) [4]. To delineate homogenized and mutually exclusive JIA subtypes, the ILAR criteria do not permit the diagnosis of ERA in subjects with psoriasis or with a first-degree relative with psoriasis, or with positive RF test, or with systemic arthritis [4].

ERA is often referred to as the pediatric counterpart of undifferentiated SpA, whereas JSpA is a broader concept including not only ERA but also a subset of juvenile psoriatic and undifferentiated arthritis, JAS, reactive arthritis, and IBD-related arthritis [22]. Compared to adult SpA, clinical axial disease is less common at disease onset in ERA and progression to JAS is unpredictable. However, when axial involvement occurs, it is often preceded by peripheral disease. Therefore, it is possible to say that ERA captures the most common presentation of SpA in childhood [15].

In the late 2000s, concerns were raised on the validity of the ILAR classification for JIA, particularly its premise of delineating uniform disease subtypes [23,24]. In light of these concerns, an international expert consensus devised by the Paediatric Rheumatology INternational Trials Organisation (PRINTO) proposed a new set of preliminary criteria for JIA geared to identify more homogeneous disease categories [25]. A prospective analysis of clinical and laboratory data is currently ongoing to validate these criteria [25]. To highlight the resemblance of ERA and adult undifferentiated SpA, the panelists agreed to rename this condition enthesitis/spondylitis-related JIA, and its definition has been partly harmonized with the adult one (Table 2). An imaging criterion, based on radiographs [26] or magnetic resonance imaging (MRI) [27] findings, has been introduced. According to the preliminary PRINTO criteria for JIA, enthesitis/spondylitis-related JIA is defined as peripheral arthritis persisting for at least 6 weeks and enthesitis, or arthritis or enthesitis, plus ≥ 3 months of inflammatory back pain (IBP) [28] and sacroiliitis on imaging, or arthritis or enthesitis plus 2 of the following: sacroiliac joint tenderness; inflammatory back pain; presence of HLA-B27 antigen; acute (symptomatic) anterior uveitis; and history of a SpA in a first-degree relative.

**Table 2:** Comparison of ILAR and provisional PRINTO criteria for the classification of ERA (adapted from refs. [4,25]).

Nomenclature	Enthesitis-Related Arthritis by ILAR Criteria	Enthesitis/Spondylitis-Related JIA by Provisional PRINTO Criteria
Inclusion criteria	Arthritis and enthesitis, or Arthritis or enthesitis with at least 2 of the following: (1) The presence of or a history of sacroiliac joint tenderness and/or inflammatory lumbosacral pain; (2) The presence of HLA-B27 antigen; (3) Onset of arthritis in a male over 6 years of age; (4) Acute (symptomatic) anterior uveitis; (5) History of ankylosing spondylitis, enthesitis-related arthritis, sacroiliitis with inflammatory bowel disease, Reiter’s syndrome, or acute anterior uveitis in a first-degree relative.	Peripheral arthritis ^1^ and enthesitis, or Arthritis or enthesitis, plus ≥ 3 months of inflammatory back pain ^2^ and sacroiliitis on imaging ^3^, or Arthritis or enthesitis plus 2 of the following: (1) Sacroiliac joint tenderness; (2) Inflammatory back pain; (3) Presence of HLA-B27 antigen; (4) Acute (symptomatic) anterior uveitis; and (5) History of a SpA in a first-degree relative.
Exclusion criteria	(1) Psoriasis or a history of psoriasis in the patient or first-degree relative; (2) The presence of IgM rheumatoid factor on at least 2 occasions at least 3 months apart; (3) The presence of systemic JIA in the patient.	None

^1^ If peripheral arthritis is present, it should persist for at least 6 weeks. ^2^ Defined according to Sieper et al. [28]. ^3^ Sacroiliitis is defined according to radiographic criteria (grade 2 bilateral radiographic sacroiliitis or grade 3) [26] or magnetic resonance imaging criteria (a. Bone marrow edema on a T2-weighted sequence sensitive for free water or bone marrow contrast enhancement on a T1-weighted sequence; b. Inflammation must be clearly present and located in a typical anatomical area, the subchondral bone; c. MRI appearance must be highly suggestive of SpA) [27]. Abbreviations: JIA: juvenile idiopathic arthritis; ILAR: International League of Associations for Rheumatology; PRINTO: Paediatric Rheumatology INternational Trials Organisation; HLA-B27: human leukocyte antigen-B27; SpA: spondyloarthritis; IgM: immunoglobulin M.

## 4. Epidemiology

ERA represents 5–30% of all JIA cases across the world [5]. Even though the oligoarticular JIA is the most common JIA subtype worldwide, in the “Epidemiology, treatment, and outcome of childhood arthritis throughout the world” (EPOCA) study, ERA was the most frequent JIA category in Southeast Asia, together with systemic JIA [5]. Indeed, a community-based cohort study showed that ERA accounts for 36% of all JIA cases in India and was the commonest JIA category (35%) in a recent single-center Chinese cohort study. Moreover, a multi-ethnic study from Canada also revealed that patients with Asian origin have a higher prevalence of ERA compared to children with European or other non-European descent [29,30]. The reasons for these findings are not clear but may be related to different genetics, epigenetics, or environmental risk factors [2]. In a study of a UK inception cohort of patients with new-onset JIA, the median age at ERA onset was 11.8 years (interquartile range 10.1–13.1) [31]. Likewise, children with ERA have a mean age at diagnosis of 10–13 years, ranging from 2.8 to 17.6 years [15]. As opposed to what is observed in JIA overall, which is more frequent in girls than in boys (ratio 2:1), ERA is prevalent in boys, with a male-to-female ratio ranging from 1,4:1 to 9:1 [2,32,33]. Why ERA is more frequent in males remains unclear. However, taking into account data on adult populations, it has been shown that sex-related dysmorphisms in immune response and genetic associations have a significant influence in the development and manifestations of autoimmune disorders [34].

## 5. Clinical Spectrum of ERA

The onset of ERA is generally insidious, even though occasionally it can be abrupt. The early symptoms are musculoskeletal pain and morning stiffness, followed by the occurrence of the signs of inflammation typical of peripheral arthritis. Patients with ERA unfrequently present systemic signs, but fatigue and a low-grade fever may occur [15].

### 5.1. Arthritis

#### 5.1.1. Peripheral Arthritis

Peripheral arthritis is described in about 90% of patients with ERA [35,36,37]. In most instances, arthritis is limited to four or fewer joints [35,36,38], although up to 25% of children may present a polyarticular involvement at onset and up to 45% throughout the course of the disease [32]. Peripheral arthritis is usually asymmetrical and mainly affects lower limbs, with knees and ankles being the most affected joints [35,36]. Tarsitis (intertarsal joints arthritis associated to the phlogosis of the overlying tendons, entheses, and soft tissues) is a typical feature of ERA, and it occurs in up to one third of patients at disease onset [39,40,41]. Tarsitis is characterized by midfoot pain, tenderness, and limitation of movement, which can lead to a forefoot adduction deformity when associated to the first metatarsophalangeal joint arthritis [15]. Unlike other JIA subtypes, hip involvement is common in ERA (20–40%) [35,36] and an isolated hip arthritis may also be the sole manifestation at disease onset [41,42]. Upper joints arthritis is infrequent, apart from the wrist involvement, which is present in about 20–25% of cases [35,36].

#### 5.1.2. Axial Involvement

Axial arthritis is less common at disease onset in ERA compared to adult SpA, in which it is often a presenting feature. Nonetheless, 30–60% of children with ERA develop sacroiliac and/or spinal inflammation within 5–10 years of disease [35,36,37,41,43,44,45,46]. However, recent studies have highlighted that axial disease could also present earlier in the course of the disease. In an Italian cohort of 59 subjects with ERA, 35% of patients reported symptoms of inflammatory back pain within 15 months after the disease onset and 29% presented sacroiliitis at the MRI [43]. Clinical signs of sacroiliitis were found in 28% of North American patients newly diagnosed with ERA included in a retrospective inception cohort study [47]. Likewise, in a multicenter cohort of 234 North American and European children with ERA, 25% of patients presented sacroiliac tenderness at the diagnosis, and an MRI confirmed a sacroiliitis in 25 out of the 45 patients undergoing imaging (55.6%) [35]. Noteworthy, Weiss and coworkers showed that active sacroiliitis by MRI is present in 20% of subjects with ERA within 6 months after diagnosis, although asymptomatic in two-third of the cases, and may be associated to radiographic signs of sclerosis or erosions [48]. As to spinal involvement, MRI may detect inflammatory or erosive lesions of the thoracolumbar spine in about 70% of patients with ERA [49,50], irrespective of the presence of clinical symptoms [50]. Altogether, those findings suggest that MRI might play an important role as a screening tool to promptly recognize axial involvement in asymptomatic children or adolescents with ERA, given the importance of the early diagnosis of axial disease to tailor appropriately the treatment [50]. Moreover, it has been shown that MRI reflects the response to treatment in adolescent ERA patients with sacroiliitis; thus, it could also be used to monitor the progression of the disease and to facilitate patient-specific therapeutic decision making [51].

The classical clinical manifestation of axial involvement is IBP [28]. IBP typically has an insidious onset, becomes worse with rest and during the night, and improves with exercise [28]. Since children may experience difficulties in describing axial symptoms, it is critical to investigate any pain in the lower back or gluteal area aggravated by periods of rest, such as prolonged sitting [41].

**Figure 1:**
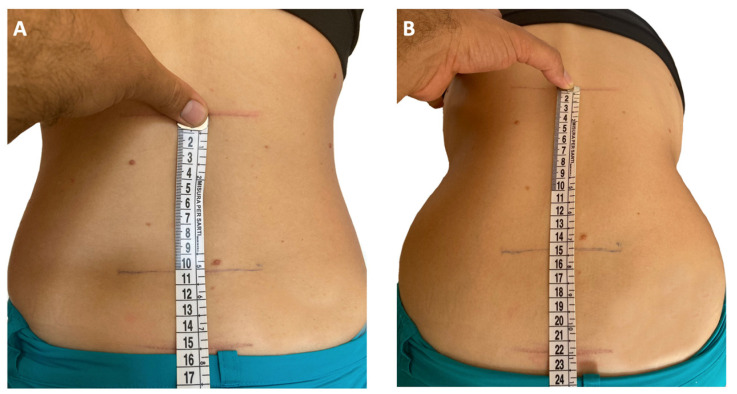
Modified Schober’s test [52]. With the patient standing upright, a line connecting the dimples of Venus is drawn as a landmark for the lumbosacral junction (**A**). Then, marks are placed midline 5 cm below (point 1) and 10 cm above lumbosacral joint (point 2). With the subject bent forward in maximum lumbar flexion with the knees straight, the distance between point 1 and point 2 is measured as an indicator of the lumbosacral spine mobility (**B**). An increase less than 6 cm indicates lumbosacral spine dysmotility [41,52].

At clinical examination, patients with sacroiliitis experience tenderness on direct compression over the sacroiliac joint. Moreover, pain may be elicited by flexion, abduction, and external rotation of the sacroiliac joints (Patrick or Faber test), or by the Gaenslen’s test [15]. When spondylitis is present, abnormalities in the profile of the back may be seen, such as a reduction of lumbar lordosis or accentuation of the thoracic kyphosis. The lumbar range of motion can be assessed through the modified Schober’s test (Figure 1) [52]. However, the results of these clinical tests should be carefully interpreted, as it has been shown that the diagnostic accuracy of the sole physical exam in the detection of axial disease in children is low [48].

Several predictors of axial involvement have been found, including HLA-B27, late onset of disease, male sex, hip arthritis, persistent elevation of inflammatory markers, and the number of active joints and entheses at the onset [43,53,54,55].

### 5.2. Enthesitis

**Figure 2:**
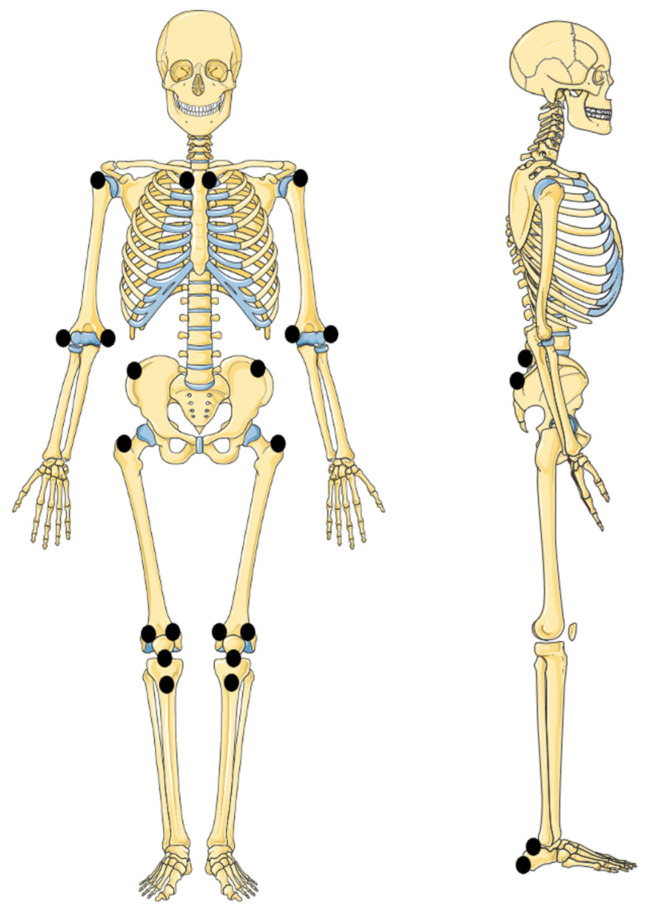
Entheses commonly explored by pediatric rheumatologists [15,41,57,60,61]. Figure modified with markings (circles) after adaptation of “Skeleton” from Servier Medical Art by Servier, licensed under a Creative Commons Attribution 3.0 Unported License.

The inflammation of entheses, which are the attachment sites of tendon, ligament, joint capsule, or fascia into bone, is a typical feature of ERA, occurring in 60–80% of patients [15,36,47,56], even though it can be less frequently observed in other JIA subtypes [56]. Enthesitis is most often symmetric and mainly involves lower limbs, especially the Achilles tendon or plantar fascia insertion to the calcaneus, quadricipital tendon attachment to the knee, and plantar fascia insertion to the metatarsal head [36,37], leading to knee, foot, or heel pain. On clinical exam, children with enthesitis present localized pain, tenderness, or swelling at the affected entheses (Table 3 and Figure 2). Although diverse tools have been validated to evaluate enthesitis’ extent and severity in adults, a standard index for enthesitis in childhood has not been developed yet and, therefore, a certain variability in the assessment of enthesitis in ERA can be observed [57,58]. Untreated enthesitis may lead to erosions, calcifications, osteopenia, and new bone formation [58]. In a large inception cohort of patients with JIA, enrolled in the Research in Arthritis in Canadian Children Emphasizing Outcomes study, children with enthesitis reported worse patient-reported outcomes than those without enthesitis, and the course of enthesitis and the course of active joint counts were associated [56,59].

**Figure 3:**
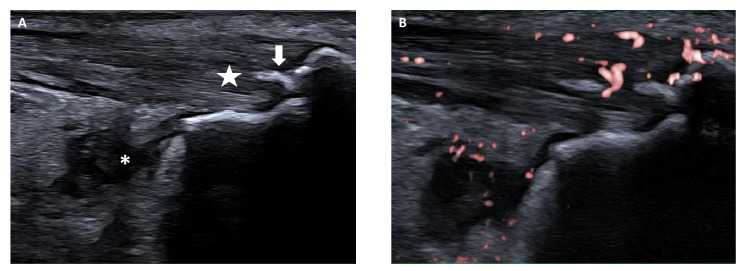
Ultrasound findings in enthesitis. Longitudinal scans of Achilles tendon and enthesis in Gray scale (**A**) and Power Doppler (**B**) show increased thickness, impairment of fibrillar pattern and hypoechoic areas (star) with active Doppler signal (**B**) within 2 mm from the bony cortex. Retrocalcaneal bursitis (asterisk) and enthesophyte (step up of the bony prominence at the end of the normal bone contour, arrow) as well as irregular contour of the calcaneal bone can also be seen as associated findings.

Ultrasonography (US) is a useful tool not only to confirm the presence of symptomatic enthesitis, but also to early detect subclinical entheseal inflammation, as shown by Shenoy et al. [37]. Recently, the Outcome Measures in Rheumatology (OMERACT) US Working Group has provided a US definition of enthesitis, i.e., hypoechoic and/or thickened insertion of the tendon close to the bone (within 2 mm from the bony cortex) which exhibits Doppler signal if active and which may show erosions and enthesophytes/calcifications as a sign of structural damage (Figure 3) [62]. Bone irregularities (bone profile changes not including definite enthesophytes nor bone erosions) and bursitis may also be present, although not included in the standardized definition of enthesitis [62]. This consensus-based definition has been devised for enthesitis in adult patients with SpA or psoriatic arthritis [62]; however, similar findings have been described in children with ERA [60]. Still, a standardized ultrasound definition of enthesitis in children is not available and the discriminant validity of the adult criteria has not been established yet [63].

### 5.3. Extra-Articular Manifestations

In contrast with the chronic asymptomatic anterior iridocyclitis associated with the ANA-positive oligoarticular or polyarticular JIA [64], ERA-related uveitis is usually an acute anterior uveitis, a non-granulomatous inflammation of the anterior segment, occurring in about 3–10% of patients [35,65,66,67]. Classically, the uveitis is unilateral, sudden, and symptomatic, with a red, painful eye, photophobia, and blurred vision, resembling the SpA-related uveitis in adulthood. Uveitis duration is generally limited, even though the inflammation can be recurrent, especially in the initial years after presentation [67]. A recent cross-sectional study, describing the features of uveitis in a large cohort of almost 4000 German patients with ERA, showed that HLA-B27 positivity and a younger age at ERA onset were independent risk factors for the development of uveitis [66]. Moreover, in the univariate analysis, patients with uveitis were more frequently male and presented higher erythrocyte sedimentation rate levels at first uveitis documentation [66]. Another relevant finding of the above-mentioned study is that, even though the course of uveitis in ERA subjects was generally comparable to HLA-B27–related uveitis in adults, in a subgroup of patients (almost 40%) the onset of uveitis was insidious or asymptomatic as in other JIA subtypes, suggesting that similar ocular screening programs may be beneficial also for ERA patients [66]. Ocular complications, such as posterior synechiae, cataract, or vitreous opacities, are relatively frequent and can be observed even at presentation [66].

The presence of gastrointestinal symptoms in a child with ERA requires a prompt evaluation to exclude the co-occurrence of an IBD. Stoll and colleagues showed that children with ERA have higher levels of calprotectin, a gut inflammation marker, compared to patients with JIA or other chronic rheumatic disorders and to healthy controls, even in the absence of gastrointestinal symptoms [68]. The same group demonstrated that subclinical signs of intestinal inflammation at the MRI enterography can be found in ERA patients with increased calprotectin [69]. The clinical significance of these findings in subjects with ERA without gastrointestinal manifestations remains unclear, even though it should be noted that one of these children was eventually diagnosed with IBD [69]. A recent study by van Straalen et al. showed that, together with a family history of autoimmunity, ERA was the strongest predictor of IBD development in patients with JIA [70].

## 6. ERA Monitoring and Outcome

In recent years, the Juvenile Arthritis Disease Activity Score (JADAS) has become the most used disease activity tool for JIA, including ERA. It is a composite disease activity score incorporating the physician global assessment of overall disease activity, the count of joints with active arthritis, the parent/patient global assessment of well-being, and an acute phase reactant [71]. Based on different methods to assess the active joint count, three versions of the JADAS were developed (JADAS10, JADAS27, and JADAS71). A reduced version of the JADAS, that lacks the acute phase reactant (clinical JADAS, cJADAS), is also available [72,73]. The patient samples used for the JADAS validation also included children with ERA [71,72]. However, the JADAS does not incorporate the evaluation of thoracic and lumbar spine and sacroiliac joints or entheses, whose involvement is one of the main features of ERA.

A specific score for juvenile SpA, named JSpA disease activity (JSpADA) index, has been devised and retrospectively validated in a cohort of 178 children with JSpA, mainly including patients with ERA [74]. JSpADA includes eight items: active joint count, active enthesis count, inflammatory markers levels, pain assessment, morning stiffness, clinical sacroiliitis, uveitis, and back mobility. The JSpADA has been recently prospectively validated in subjects with ERA, showing good measurement properties [75,76]. Srinivasalu and coworkers also validated a shorter version of the JSpADA, i.e., modified JSpADA, not incorporating the inflammatory markers and the assessment of back mobility. This six-element score, which exhibited good face validity, discriminant ability, and responsivess to change, was devised with the aim of making the JSpADA simpler to use, thus implementing its utilization in both clinical practice and research trials [75].

Some scores for adult SpA disease activity assessment have also been validated for ERA, perfoming well in terms of clinimetric properties [76].

With regard to ERA outcome, it has been shown that ERA is associated with higher pain intensity, worse functional status, and poorer health status compared to other JIA categories [32,77,78,79], and that up to 40% of patients may eventually develop AS, even though the rate of progression to severe axial disease with ankylosis remains unclear [54,80]. However, some of these findings date back to the “pre-biologics era” and it is possible that the early administration of biological agents could have altered the disease course. In fact, in a recent retrospective cohort study by Deligeorgakis and collaeagues aimed at describing the profile of ERA patients in the era of biologics, the early administration of biological drugs and a good treatment adherence were associated with improved long-term outcomes [81].

Several predictors of poor outcome have been found, including hip involvement, tarsitis, older age at onset, enthesitis, sacroiliitis, and HLA-B27 positivity [58,59,82,83,84]. In a recent study assessing the clinical and genetic predictors of the long-term functional outcome in 181 patients with ERA, the delay of diagnosis was the sole predictor of poor functional status and tarsitis was the sole predictor of persistent disease activity at the multivariate analysis [85].

## 7. ERA Treatment

### 7.1. Current Therapeutic Recommendations

As recommended in the current ACR guidelines for the treatment of JIA, ERA management includes pharmacological and nonpharmacological measures depending on several factors, such as the number of active joints, the presence of enthesitis, and the occurrence of axial disease [86,87,88]. According to the international treat-to-target recommendations for JIA published in 2018, therapeutic interventions are aimed to control the disease activity; to prevent joint damage; to avoid comorbidities and medication toxicities; and to optimize functional status, growth and development, quality of life, and social participation [89].

Among nonpharmacological interventions, physical exercises emphasizing strength, flexibility, and balance are associated with enhancements in functional activity, body structure, and quality of life in children with JIA, while occupational therapy promotes increased participation in activities of daily living and family routines [86,90].

The pharmacological options for ERA treatment include nonsteroidal anti-inflammatory drugs (NSAIDs), glucocorticoids, conventional synthetic disease-modifying antirheumatic drugs (csDMARDs) including sulfasalazine (SSZ) and methotrexate (MTX), and biologic DMARDs (bDMARDs, Table 4).

In the case of isolated peripheral arthritis without axial disease or enthesitis, the therapeutic strategy depends upon the number of active joints or the presence of poor prognostic features, as for the other non-systemic JIA subtypes (Table 5). Intra-articular glucocorticoids and NSAIDs are advised as first-line therapy when less than five joints are involved (oligoarthritis population), and as adjunct therapy for peripheral arthritis affecting five or more joints (polyarthritis population). csDMARDs should be initiated in the instance of poor response to NSAIDs or intra-articular glucocorticoids in oligoarthritis and as an initial therapy in polyarthritis. In such cases, MTX is indicated as the preferable csDMARD. bDMARDs are recommended in patients not responsive to (or intolerant of) csDMARDs and, as part of first-line therapy, in polyarthritis with high disease activity or poor prognostic features [87,88].

The occurrence of sacroiliitis or enthesitis changes the therapeutic approach. The recommended first-line therapy is NSAIDs in both cases, but MTX is not the treatment of choice if NSAIDs are ineffective (or not tolerated). It has been shown that MTX is ineffective in the treatment of axial disease in adults [94,95]; therefore, even though similar trials have not been performed in children, the ACR guidelines for JIA strongly recommend against MTX monotherapy in children or adolescents with active sacroiliitis despite NSAIDs, based on extrapolation data and clinical experience [88,96]. In such cases, adding a Tumor Necrosis Factor-α inhibitor (TNFi) is strongly advised. MTX may be used as additional treatment in patients with co-occurrent peripheral polyarthritis or to prevent the development of neutralizing antidrug antibodies against monoclonal TNFi. SSZ may be used in patients with sacroiliitis presenting contraindications to TNFi or having failed more than one TNFi [88]. Similarly, the role of MTX is limited in the management of enthesitis: in case of active enthesitis not being responsive to NSAIDs, using a TNFi is conditionally recommended over MTX or SSZ. A trial of csDMARDs can be considered for subjects with enthesitis having contraindications to TNFi or concomitant active peripheral polyarthritis [88]. The effectiveness of TNFi in the treatment of ERA has been proven not only by retrospective analysis [55,97] but also by multiple randomized controlled trials [98,99,100,101,102,103]. Among TNFi, Etanercept is the most widely used in daily practice, while adalimumab is preferred in patients with concomitant uveitis.

In patients with polyarthritis, sacroiliitis, and/or enthesitis, a short course of oral glucocorticoids (<3 months) may be considered as bridge therapy while stepping-up the therapy, and intra-articular glucocorticoid injection may be useful as an adjunct therapy [88].

### 7.2. Emerging Biological Agents and Small Molecules

In recent decades, several studies have shown that the interleukin (IL)-17/IL-23 axis plays a pivotal role in the pathogenesis of JIA [104,105,106]. It has been shown that the synovial fluid of children with ERA present high levels of IL-17, in correlation with the disease activity, and a predominant representation of the T helper 17 lymphocyte population [107,108]. These findings highlighted the relevance of the IL-23/IL-17 axis as a potential treatment target. Some drugs targeting this pathway (Table 4) have demonstrated a robust therapeutic effect in adult SpA [109,110,111]. The Food and Drug Administration (FDA) and the European Medicines Agency (EMA) have recently approved a monoclonal antibody against IL-17A, secukinumab, for ERA and JPsA treatment in case of insufficient response to (or intolerance of) conventional therapy, based on data from the Phase III JUNIPERA trial. In this randomized controlled trial, secukinumab was more effective in increasing time to time to disease relapse than placebo and exhibited a good safety profile in a cohort of 86 children and adolescents with active ERA or JPsA [112]. Moreover, a recent retrospective monocentric study revealed that secukinumab was able to reduce the JSpADA and the JADAS10 scores in patients with ERA not responsive to TNFi [113]. Ixekizumab, another anti-IL-17A agent, has already been approved for the treatment of axial SpA in adulthood, and its effectiveness and safety in children with ERA and JPsA are being currently investigating in a multicenter, open-label trial (ClinicalTrials.gov Identifier: NCT04527380). A case series of another drug blocking the IL-17/IL-23 axis, ustekinumab, in five adolescents with ERA refractory to TNFi, noted an improvement in active enthesitis and arthritis [114].

In the last few years, small molecules, such as the inhibitors of Janus kinase/signal transducer and activator of transcription (JAK/STAT) pathway, have been developed and tested for the treatment of chronic rheumatic diseases, showing promising results. The efficacy of tofacitinib, an oral JAK inhibitor, has been demonstrated in a pivotal trial conducted on a group of patients with a polyarticular course JIA, including 21 subjects with ERA [115]. This molecule has been recently approved for the treatment of active polyarticular course JIA by FDA and for the treatment of both polyarticular JIA and JPsA by EMA. In a recent randomized clinical phase 3 trial, another JAK inhibitor, baricitinib, has demonstrated therapeutic effectiveness with an acceptable safety profile in a cohort of 220 JIA patients with inadequate response or intolerance to standard therapy, including 50 subjects with ERA [116].

## 8. Conclusions

ERA is a form of childhood chronic arthritis, accounting for 5–30% of all JIA cases worldwide. ERA is currently defined according to the ILAR classification criteria for JIA; however, the international network PRINTO has recently proposed new provisional criteria for ERA, whose validation is currently ongoing, as part of a wider revision of the ILAR criteria. ERA shares some key clinical features with adult SpA, including peripheral arthritis, axial involvement, enthesitis, anterior acute uveitis, and association with HLA-B27. The axial involvement is less frequent in ERA compared to adult SpA, even though the increased use of MRI has shown that a considerable proportion of patients with ERA present a subclinical axial disease. With regard to entheseal inflammation, there is the need of a standardized and validated US definition for enthesitis in children, which is currently lacking. Diverse instruments can be used to monitor the disease activity of ERA, including the JADAS in its different versions, the JSpADA, and the modified JSpADA. The therapeutic recommendations for ERA do not differ from those used in other non-systemic JIA subtypes, unless sacroiliitis and/or enthesitis are present. In such cases, the early use of a TNFi should be promptly considered. Novel therapeutic agents are emerging, and hold promise for the treatment of ERA, including IL-17/IL-23 or JAK/STAT pathways blockers.

## Figures and Tables

**Table 1:** Frequency of the ILAR JIA subtypes across the world. Data from Ref. [5].

JIA Subtypes Defined by ILAR	Frequency ^1^
Systemic arthritis	4.2–33%
Oligoarthritis	10.8–56.7%
Polyarthritis (Rheumatoid Factor positive)	1.3–11.2%
Polyarthritis (Rheumatoid Factor negative)	12.7–31.5%
Psoriatic arthritis	1.3–7.1%
Enthesitis-related arthritis	5.4–29.8%
Undifferentiated arthritis	3.7–10.4%

^1^ The indicated frequencies are extracted from Ref. [5] and refer to the percentage of all JIA subjects from different geographic areas enrolled in the “Epidemiology, treatment, and outcome of childhood arthritis throughout the world” study. Abbreviations: JIA: juvenile idiopathic arthritis. ILAR: International League of Associations for Rheumatology.

**Table 3:** Anatomical sites for the evaluation of entheses in JIA. Modified from Refs. [15,41] from Elsevier publisher, with re-use permitted according to the STM (Int’l Assoc. of Scientific, Technical & Medical Publishers) Permissions Guidelines.

Anatomic Region	Sites for Entheseal Assessment
Chest	First and seventh costosternal junctions
Upper extremity	Supraspinatus attachment into greater tuberosity of humerus
	Common flexor attachment at medial epicondyle of humerus Common extensor attachment at lateral epicondyle of humerus
Spine	Fifth lumbar spinous process
Pelvis	Abdominal muscle attachments to iliac crest
	Sartorius attachment at anterior superior iliac spine
	Posterior superior iliac spine
	Hip extensor attachment at greater trochanter of femur
	Gracilis and adduction attachment to pubis symphysis
	Hamstrings attachment to ischial tuberosity
Knee	Quadriceps tendon attachment to patella (2 and 10 o’clock)
	Infrapatellar ligament attachment to patella (6 o’clock) and tibial tuberosity
Foot and ankle	Achilles tendon attachment to calcaneus
	Plantar fascia attachment to calcaneus
	Plantar fascia attachment to metatarsal heads
	Plantar fascia attachment to base of fifth metatarsal

**Table 4:** Biological agents mainly used or studied in ERA and their mechanism of action [91,92,93].

bDMARDs	Composition and Action Mechanism
Etanercept	TNF-α receptor fusion protein able to bind circulating TNF-α
Adalimumab	Humanized monoclonal antibody binding both soluble and membrane-bound TNF-α
Infliximab	Chimeric monoclonal antibody binding both soluble and membrane-bound TNF-α
Golimumab	Human monoclonal antibody binding both soluble and membrane-bound TNF-α
Secukimumab	Human monoclonal antibody that binds IL-17A, avoiding the activation of IL-17 pathway
Ustekimumab	Human monoclonal antibody directed against the p40 subunit shared by IL-12 and IL-23, preventing the Th-17 cells activation
Ixekizumab	Humanized monoclonal antibody targeting IL-17A, avoiding the activation of IL-17 pathway
Tofacitinib	JAK1/JAK3 inhibitor able to prevent JAK-STAT cascade activation
Baricitinib	JAK1/JAK2 inhibitor able to prevent JAK-STAT cascade activation

ERA, enthesitis related arthritis; TNF-α, Tumor necrosis factor alpha; IL, interleukin; Th, T helper; JAK, Janus kinase; STAT, Signal transducer and activator of transcription.

**Table 5:** Therapeutic approach for ERA, based on current American College of Rheumatologic guidelines for the treatment of JIA [87,88].

	Isolated Peripheral Arthritis (<5 Affected Joints)	Isolated Peripheral Arthritis (≥5 Affected Joints) *	Presence of Sacroiliitis *	Presence of Enthesitis *
First-line therapy	IAGCs, trial of NSAIDs	csDMARD with MTX as preferred agent IAGCs and NSAIDs as adjunct therapy If poor prognostic features ** are present, consider a bDMARD	NSAIDs	NSAIDs
Inadequate responseto first-line therapy	csDMARD, with MTX as preferred agent	Add a bDMARD in patient treated with a csDMARD Switch to another bDMARD if the patient is already receiving a bDMARD	Add a TNFi SSZ for patients having contraindications to TNFi IAGC of the sacroiliac joints conditionally recommended	TNFi A trial of MTX or SSZ may be considered for patients having contraindications to TNFi, or mild enthesitis, or co-occurrent active peripheral polyarthritis
Inadequate responseto second-line therapy	bDMARD	Switch to another bDMARD	Switch to another TNFi SSZ for patients having failed more than one TNFi	

ERA, enthesitis related arthritis; JIA: juvenile idiopathic arthritis; IAGCs, Intra-articular glucocorticoids; NSAIDs, nonsteroidal anti-inflammatory drugs; csDMARD, conventional synthetic disease-modifying antirheumatic drug; MTX, methotrexate; SSZ, sulfasalazine; bDMARD, biological disease-modifying antirheumatic drugs; TNFi, Tumor Necrosis Factor-α inhibitor. * In patients with polyarthritis, sacroiliitis, and/or enthesitis, a short course of oral glucocorticoids (<3 months) may be used as a bridging treatment while initiating or escalating the therapy. ** Features of poor outcome include high disease activity, involvement of high risk-joints (e.g., cervical spine, ankle, wrist, temporomandibular joint, or hip), presence or high risk of joint damage. Consideration of poor prognostic features should also guide treatment decision in oligoarthritis.

## Data Availability

Not applicable.
